# Electrical conductivity-based contrast imaging for characterizing prostatic tissues: in vivo animal feasibility study

**DOI:** 10.1186/s12894-019-0532-y

**Published:** 2019-10-21

**Authors:** Yong Soo Cho, Young Hoe Hur, Hyun Ju Seon, Jin Woong Kim, Hyung Joong Kim

**Affiliations:** 1 Department of Radiology, Chosun University Hospital and Chosun University College of Medicine, 365 Pilmun-daero, Dong-gu, Gwangju, 61453 South Korea; 2 Department of Hepato-Biliary-Pancreas Surgery, Chonnam National University Hwasun Hospital and Chonnam National University Medical School, Gwangju, 61469 South Korea; 3 Department of Biomedical Engineering, Kyung Hee University, 23 Kyungheedaero, Dongdaemungu, Seoul, 02447 South Korea

**Keywords:** Electrical conductivity, Magnetic resonance imaging, Prostate, Central zone, Peripheral zone

## Abstract

**Background:**

Electrical conductivity-based magnetic resonance (MR) imaging may provide unique information on tissue condition because its contrast originates from the concentration and mobility of ions in the cellular space. We imaged the conductivity of normal canine prostate in vivo and evaluated tissue contrast in terms of both the conductivity distribution and anatomical significance.

**Methods:**

Five healthy laboratory beagles were used. After clipping the pelvis hair, we attached electrodes and placed each dog inside the bore of an MRI scanner. During MR scanning, we injected imaging currents into two mutually orthogonal directions between two pairs of electrodes. A multi spin echo pulse sequence was used to obtain the MR magnitude and magnetic flux density images. The projected current density algorithm was used to reconstruct the conductivity image.

**Results:**

Conductivity images showed unique contrast depending on the prostatic tissues. From the conductivity distribution, conductivity was highest in the center area and lower in the order of the middle and outer areas of prostatic tissues. The middle and outer areas were, respectively, 11.2 and 25.5% lower than the center area. Considering anatomical significance, conductivity was highest in the central zone and lower in the order of the transitional and peripheral zones in all prostates. The transitional and peripheral zones were, respectively, 7.5 and 17.8% lower than the central zone.

**Conclusions:**

Current conductivity-based MR imaging can differentiate prostatic tissues without using any contrast media or additional MR scans. The electrical conductivity images with unique contrast to tissue condition can provide a prior information on tissues in situ to be used for human imaging.

## Background

Prostate cancer and benign prostatic hyperplasia are the most common diseases and significant causes of death for older adult men [1]. The morphological and functional examinations are widely used to characterize the tissue properties of prostatic diseases [2, 3]. The prostate gland can be divided into the central area and peripheral area [4]. The central area consists of the central and transitional zones. The peripheral area consists of the peripheral zone and a small amount of anterior fibromuscular stroma without glandular tissue. There exist differences in volume, tissue composition, and incidence of prostatic diseases among the three zones [4, 5]. Specically, the incidence of prostate cancers and prostatitis is higher in the peripheral zone than in the other zones. Therefore, image-based qualitative and quantitative information on prostatic tissues can provide a prior knowledge of the zonal information with prostatic diseases.

The prostate is an imaging area of growing concern related with aging. Although prostate cancer is a common cancer in older adult men, imaging diagnosis in detection and localization of prostate cancer remains challenging compared with other abdominopelvic cancers [6]. Among imaging modalities, magnetic resonance imaging (MRI) is a powerful tool for visualizing tissue conditions because of its excellent soft tissue contrast by various imaging parameters [7]. In general, T2-weighted MR imaging provides enough contrast on zonal information, but tumor localization is limited and prostate biopsy is mandatory to diagnose prostate cancer. Recently, the introduction of a multi-parametric MR imaging method, which is a combination of dynamic contrast-enhanced MRI and diffusion-weighted imaging and MR spectroscopy, has improved the diagnostic accuracy in prostatic diseases [6]. The multi-parametric MR imaging is used for assessing intraprostatic diseases, while positron emission tomography (PET) has advantage in the detection of extraprostatic diseases [8]. Despite these technical advances, there still exist difficulties in tumor diagnosis and the assessment of treatment response after hormonal or radiation therapy. Thus, prostate biopsy is performed in almost all patients with suspected prostate cancer [6, 9]. New imaging techniques are being studied to improve the diagnosis and monitor the treatment response in patients with prostate cancer.

MR-based tissue property mapping is an emerging technique that uses an MR scanner to obtain non-invasive information concerning electrical tissue properties such as conductivity and permittivity [10–12]. Such electrical tissue properties provide alternative information on tissue structure and function, and can serve as a good complement to the information provided by traditional MRI methods [13–15]. Several studies reported the electrical properties of the prostatic tissues by measuring its values in vitro or ex vivo [16], but no in vivo imaging study can non-invasively provide information on the tissue condition in situ. The recent magnetic resonance electrical impedance tomography (MREIT) method enables high-resolution imaging of electrical conductivity by the externally injected currents [12, 14, 17]. The electrical conductivity of biological tissues is primarily determined by the concentration and mobility of ions that exist in the intra- and extracellular structures [17, 18]. The electrical conductivity contrast is closely related to the structural and functional conditions of tissues and organs [11, 12, 17, 18]. Therefore, the electrical conductivity has potential to provide direct information on the microscopic structures that can be represented as a macroscopic images with novel contrast.

The purpose of this study was to show the feasibility of in vivo conductivity imaging of the prostate, which can provide novel contrast on the tissue condition relating to the zonal information. From the normal canine prostate, we imaged in vivo electrical conductivity of prostatic tissues and measured its values in terms of both the conductivity distribution and anatomical significance to quantitatively distinguish the tissue condition.

## Methods

### Animal preparation

**Fig. 1:**
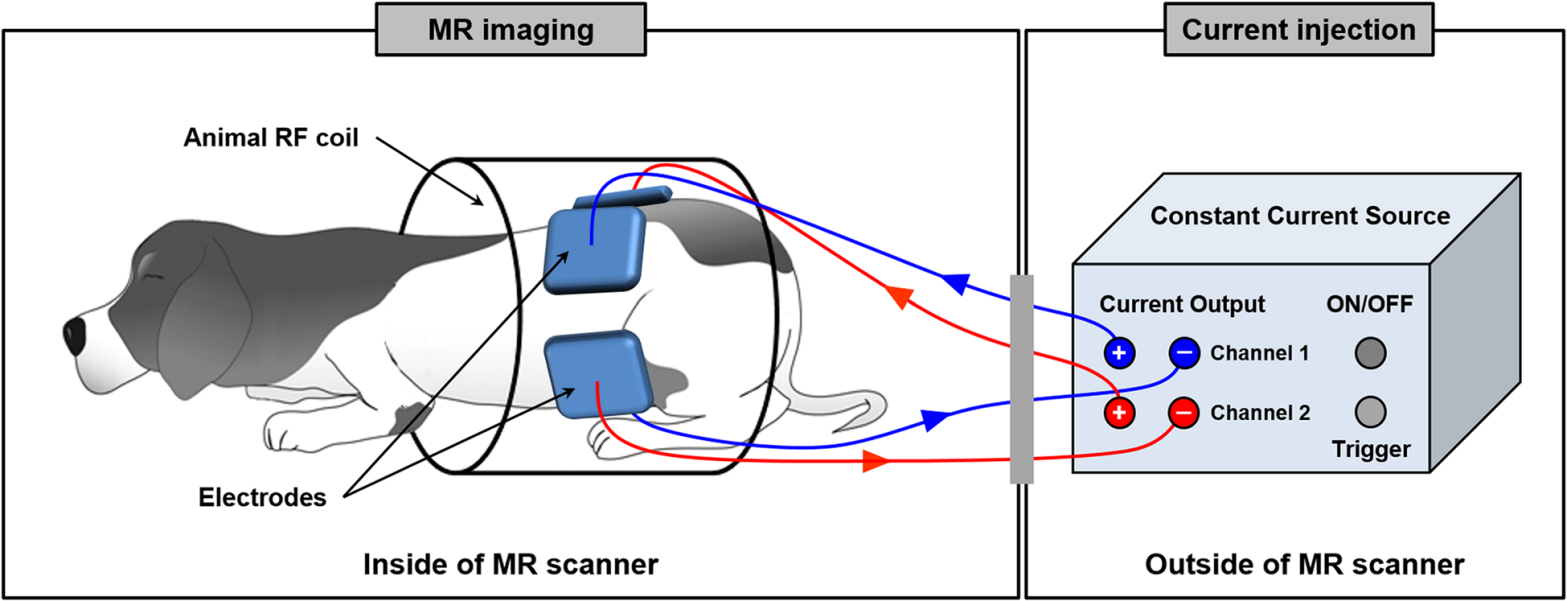
Experimental setup for electrical conductivity-based MR imaging in a canine pelvis. The imaging objects are located inside the MR scanner and the imaging current is injected into the pelvis through the electrodes during MR scanning

Five healthy laboratory beagles (2–5 years old, weighing 5–13 kg; Harlan Interfauna, Huntingdon, Cambridgeshire, UK) were used for imaging experiments. All experimental protocols were performed in accordance with the regulations of the Institutional Animal Care and Use Committee (IACUC), and were approved by the Ethics Committee of Kyung Hee University, Korea (No. KHUASP-14-25). All were healthy without a history of any known disease or signs of metabolic and neurological problems. We injected 0.1 mg/kg of atropine sulfate to prevent the dog from salivating during the experiments. Ten minutes later, we anesthetized the dog with an intramuscular injection of 0.2 ml/kg Tiletamine and Zolazepam (Zoletil 50, Virbac, France). After clipping and shaving the hair at four locations on the pelvis, we attached four carbon-hydrogel electrodes (HUREV Co. Ltd., Korea) and placed the dog inside the bore of a 3 T MRI scanner (Achieva TX, Philips, Amsterdam, the Netherlands) (Fig. 1). Inside the MR scanner, we intubated the dog using an endotracheal tube and began general anesthesia using a veterinary anesthesia system (VME, MATRX, USA). We used 2% isoflurane mixed with oxygen at 800 ml/min flow rate. Ventilation was machine-controlled using a ventilator (M-2002, Hallowell EMC, USA) at a respiration rate of 15 bpm and tidal volume of 200 ml.

### Imaging experiment

Anatomical T1-weighted, T2-weighted MR images were obtained to confirm the morphological information of the canine pelvis. For the electrical conductivity images of the in vivo prostate, a multi-echo spin echo pulse sequence was used to simultaneously obtain the MR magnitude and magnetic flux density (*B*_*z*_) images [17]. The imaging parameters were as follows; TR/TE = 900/15, 30, 45 ms (3 echoes), FOV = 220 × 220 mm^2^, slice thickness = 3 mm, NEX = 8, matrix size = 128 × 128, number of slices = 6, and total imaging time = 40 min. During MR scanning, the first current *I*_1_ was injected between one opposing pair of electrodes using a constant current source. The injected current was 5 mA amplitude and a total pulse width of 81 ms. After acquiring the first magnetic flux density (*B*_*z*_) dataset for *I*_1_, the second injection current *I*_2_ with the same amplitude and pulse width was injected through the other pair of opposing electrodes to obtain the second dataset. After imaging experiments, dogs were euthanized with sodium pentobarbital (80 mg/kg IV; Entobar Injection, Hanrim Pharm, Gyeonggi, South Korea) [19].

### Conductivity image reconstruction and data analysis

The raw data was extracted from the MR spectrometer after finishing the imaging experiments. To reconstruct electrical conductivity images of the canine pelvis, the magnetic flux densities were generated by removing the effects of injected current at raw datasets. Specifically, the magnetic flux density information was determined by subtracting the two datasets with positive and negative currents. And the final low-frequency conductivity was reconstructed from the measured magnetic flux density by applying the projected current density method [20]. The detailed image reconstruction for conductivity was described in the work of Sajib et al [21]. The quantitative analysis was performed on the results from three dogs which covered the entire prostatic tissues such as the central, transitional, and peripheral zones. The regions-of-interest (ROIs) were positioned at several prostatic tissues by an experienced radiologist and their values were measured from both the signal change in conductivity distribution (Fig. 4a) and anatomical significance in T2-weighted MR images (Fig. 5a). All values were expressed as means ± standard deviations, and plotted as bar graphs to compare between different prostatic zones.

## Results

### Electrical conductivity images of prostate

**Fig. 2:**
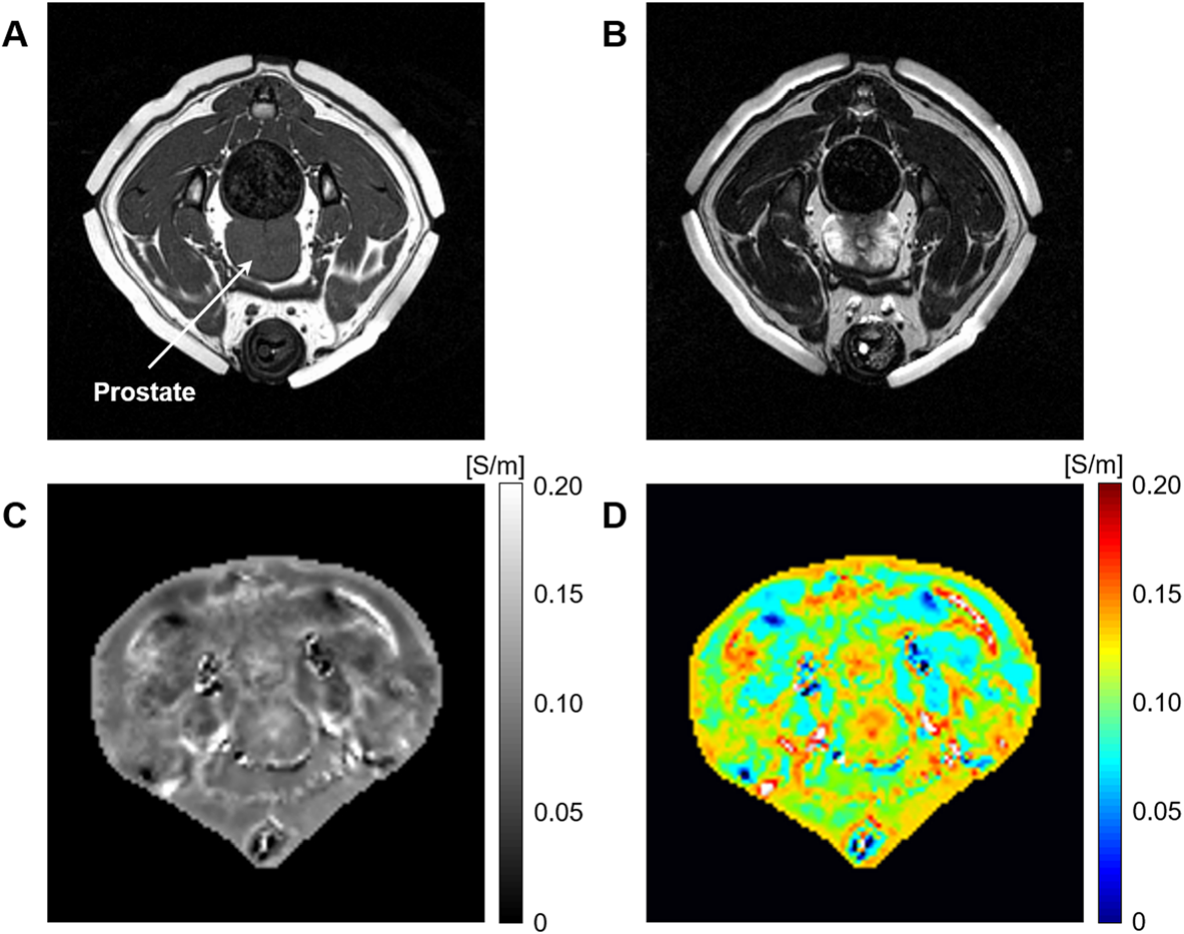
Typical results of electrical conductivity imaging in a canine pelvis. **a** T1-weighted MR image, **b** T2-weighted MR image, **c** reconstructed conductivity image, and **d** pseudo color image of pelvis

Figure 2 shows typical results of electrical conductivity-based MR imaging from an in vivo canine pelvis. The T1- and T2-weighted MR images (Fig. 2a and b) indicate the anatomical structures and MR tissue contrast of the canine pelvis. The electrical conductivity and its pseudo color images (Fig. 2c and d) show different conductivity contrasts of pelvis regions, including muscles, bones, and adipose tissues. When we focused on the prostate, there was no tissue contrast in the T1-weighted image, but the T2-weighted and electrical conductivity images showed clear contrast between the prostatic tissues. Specifically, the signal intensity of the peripheral area was the highest in the T2-weighted image, but the lowest in the electrical conductivity image. On the contrary, the signal intensity of the central area was the lowest in the T2-weighted image, but the highest in the electrical conductivity image.

**Fig. 3:**
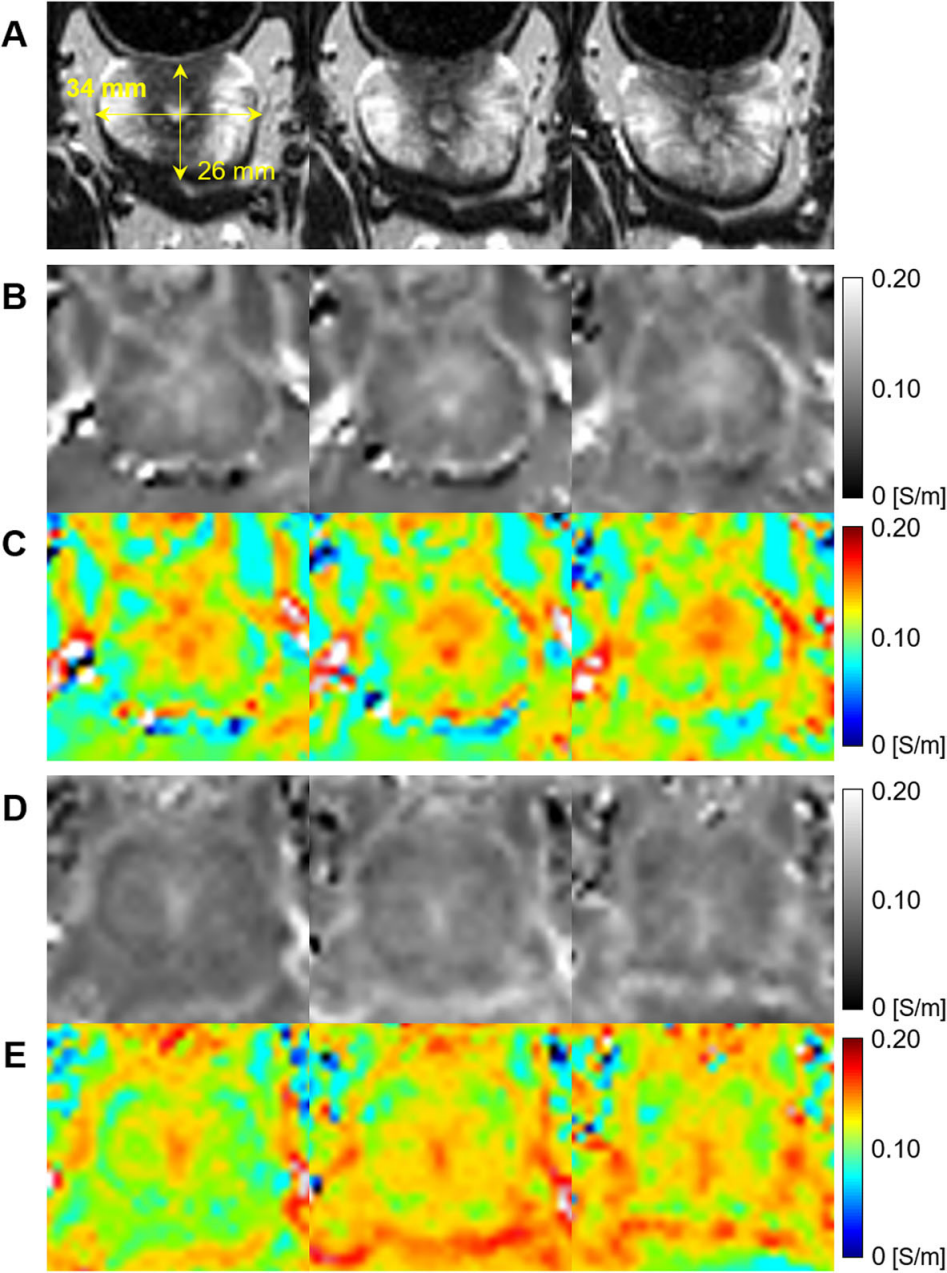
Resulting conductivity images in three continuous slices from two canine prostates. **a** T2-weighted MR images, **b** and **c** reconstructed conductivity and pseudo color images of the first prostate, **d** and **e** images from the second prostate

Figure 3 shows the electrical conductivity images with three continuous slices from two canine prostates. The anatomical structure and size of the prostate can be confirmed from the T2-weighted MR images (Fig. 3a). The electrical conductivity (Fig. 3b and d) and its pseudo color images (Fig. 3c and e) of two prostates showed the unique contrast between the prostatic tissues. The conductivity of the central area was higher than that of the peripheral area in all slices.

### Prostatic tissue analysis by electrical conductivity

**Fig. 4:**
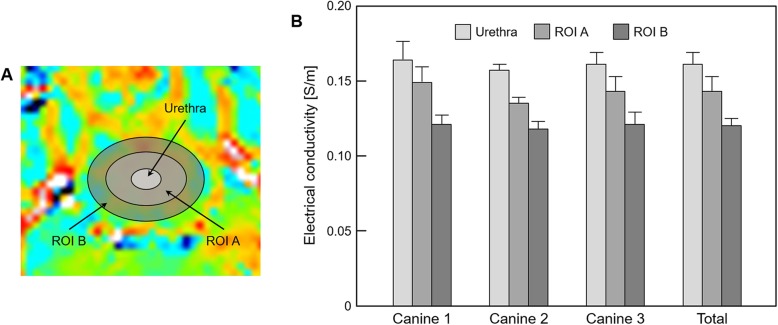
Bar graph showing the comparison of prostatic tissues based on the contrast changes in electrical conductivity images. **a** ROIs are located in three prostatic tissues, **b** electrical conductivity is measured from the corresponding regions

Figure 4 and Table 1 represent the quantitative analysis of prostatic tissues based on the contrast changes in electrical conductivity images. The regions-of-interest (ROIs) were located and confirmed by an experienced radiologist at three regions, indicating the urethra, ROI A, and ROI B, which are marked in Fig. 4a. Most of ROI A covered the central area, while ROI B was the peripheral area. From the results of three prostates, the conductivity was the highest in the urethra and lower in the order of ROI A and ROI B. Specifically, ROI A was about 11.2% and ROI B was about 25.5% lower than the conductivity of the urethra (Table 1).

**Table 1:** Measurement of electrical conductivity of prostatic tissues based on the conductivity distribution

Conductivity [S/m]	Urethra	ROI A	ROI B
Canine 1	0.164 ± 0.012	0.149 ± 0.010, (9.1%)^a^	0.121 ± 0.006, (26.2%)^a^
Canine 2	0.157 ± 0.004	0.135 ± 0.004, (14.0%)	0.118 ± 0.005, (24.8%)
Canine 3	0.161 ± 0.008	0.143 ± 0.010, (11.2%)	0.121 ± 0.008, (24.8%)
Average	0.161 ± 0.008	0.143 ± 0.010, (11.2%)	0.120 ± 0.005, (25.5%)

Values are presented as mean ± standard deviation

^a^Parentheses indicate the percentage changes based on the conductivity of the urethra

**Fig. 5:**
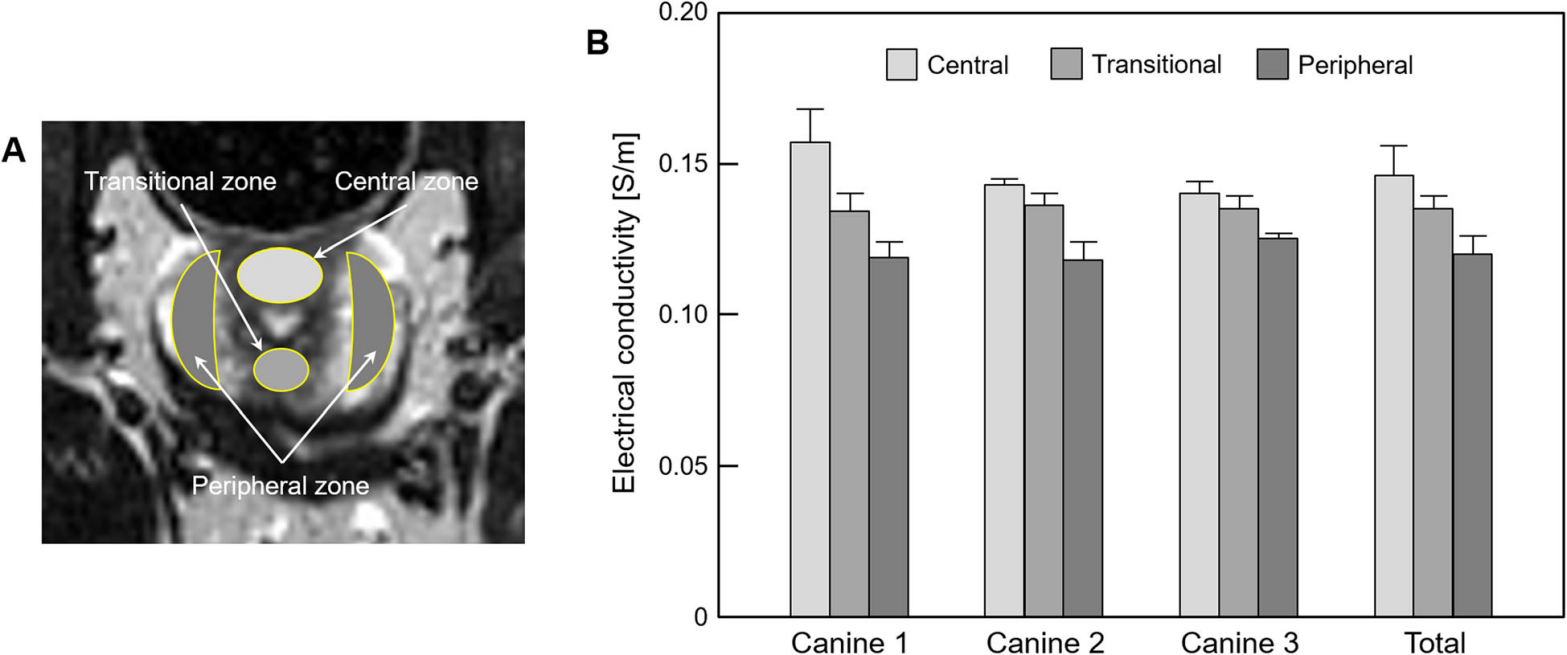
Bar graph showing the comparison of prostatic tissues based on the anatomical significance in the T2-weighted MR image. **a** ROIs are located in three anatomical regions of the prostate, **b** electrical conductivity is measured from the corresponding regions

Figure 5 and Table 2 represent the quantitative analysis of prostatic tissues based on the anatomical significance in the T2-weighted MR image. The ROIs were located and confirmed by an experienced radiologist at three zones, indicating the central, transitional, and peripheral zones, which are marked in Fig. 5a. The conductivity was the highest in the central zone, while it was the lowest in the peripheral zone in all prostates. Specifically, the transitional zone was about 7.5% and peripheral zone was about 17.8% lower than the conductivity of the central zone (Table 2).

**Table 2:** Measurement of electrical conductivity of prostatic tissues based on the anatomical significance

Conductivity [S/m]	Central zone	Transitional zone	Peripheral zone
Canine 1	0.157 ± 0.011	0.134 ± 0.006, (14.6%)^a^	0.119 ± 0.005, (24.2%)^a^
Canine 2	0.143 ± 0.002	0.136 ± 0.004, (4.9%)	0.118 ± 0.006, (17.5%)
Canine 3	0.140 ± 0.004	0.135 ± 0.004, (3.6%)	0.125 ± 0.002, (10.7%)
Average	0.146 ± 0.010	0.135 ± 0.004, (7.5%)	0.120 ± 0.006, (17.8%)

Values are presented as mean ± standard deviation

^a^Parentheses indicate the percentage changes based on the conductivity of the central zone

## Discussion

Differences exist in the tissue composition of the various prostate zones, such as the gland (acini) density per zone, and the incidence of prostatic diseases are known to have zonal predilections [5]. The water-rich duct and acini within the peripheral zone are similar to that of central zone. However, the peripheral zone has a larger extracellular space than the central zone because the density of the stroma, which connects between the duct and acini, is lower in the peripheral zone than in the central zone [4, 5]. Moreover, prostate cancer has a higher density of glandular elements and lesser space of mucin or fluid than a normal prostate gland and benign prostate hyperplasia. This difference in histologic composition makes prostate cancer different from a normal prostate gland and benign prostate hyperplasia in the prostate. The difference is even more pronounced in the peripheral zone. This cellular environment is one factor for determining electrical tissue conductivity that can be represented as a macroscopic image with novel contrast [18, 20, 21].

Since the electrical tissue conductivity basically originates from the concentration and mobility of ions in the extra- and intracellular space, image contrast can be interpreted as different tissue conditions in terms of bio-electromagnetics. From the results of the conductivity distribution in prostatic tissues, the conductivity was decreased in the order of the urethra, ROI A, and ROI B. The high conductivity of the urethra originates from the urethra itself and a certain amount of urine, which contains many electrolytes. ROI A and B roughly correspond to the central and peripheral areas of the prostate, respectively. The conductivity of the central area, which consists of central and transition zones, was higher than that of the peripheral area, mostly including the peripheral zone. This is consistent with the difference in conductivity due to the anatomical significances.

From the anatomical zonal analysis, the conductivity was decreased in the order of the central, transitional, and peripheral zones. The major difference between the prostatic zones is the volume of glandular tissue and density of connective tissues, such as the stroma. The central and transitional zones occupy 25% of the volume of the glandular tissue and the peripheral zone occupies roughly 75% [22–24]. The density of the stroma is higher in the central and transitional zones than in the peripheral zone [22–24]. There has been no in vivo measurement study of prostate tissues, but the electrical conductivity of glandular tissue and the stroma have been reported as 0.097 and 0.120 S/m, respectively, at low-frequency by in vitro measurements [16, 25, 26]. Although there exists a difference between in vivo and in vitro measurements, the low conductivity of the peripheral zone can be explained from these results. The histological characteristics between the central and transitional zones are almost similar, with the only difference being the volume, location, and tissue composition ratio [5]. However, our results show that there was a difference in conductivity between the two zones. From the viewpoint of the bio-electromagnetism, the transitional zone may have an intermediate tissue condition between the central and peripheral zones, but a detailed comparison should be addressed in future studies.

In vivo electrical conductivity imaging (ECI) could provide alternative information on tissue structure and function using electrical tissue properties such as conductivity and permittivity. Electrical conductivity is an inherent physical property of living tissues and it provides absolute values [11, 17, 18]. Therefore, the tissue condition can be quantified and discriminated on the basis of absolute conductivity values. These preliminary results suggest the possibility of ECI as a new imaging biomarker for prostate disease. ECI may serve as a good complement to current prostate imaging in diagnosing prostate disease and assessing treatment response, especially for prostate cancer. Moreover, ECI can provide immediate information on tissue anisotropy. When ECI is used with current multiparametric MR imaging, it may be helpful in the diagnosis of prostatic cancer, such as in localization and characterization, and in the assessment of the treatment response in the early period.

We acknowledge that this study has several limitations. First, the relatively long acquisition time and poor spatial resolution should be addressed for clinical applications. Recent advances in ECI technique, such as conductivity tensor imaging (CTI), have demonstrated the potential to improve current conductivity imaging methods in terms of acquisition time and spatial resolution [27]. In addition, the high sensitivity of ECI to tissue response following radiation therapy (RT) may reveal the possibility of ECI for clinical application in prostate diseases [28]. Second, the current study was performed on a small number of normal animal prostates, thus, further study should be performed with large numbers of normal prostates for statistical analysis, animal disease models such as prostate cancer or benign prostate hyperplasia, and human prostates. Third, the lack of accurate correlation between tissue histology and the value of ECI according to ROI and zonal anatomy might be problematic. However, regarding the well established zonal anatomy on T2-weighted imaging in the current study, ECI could provide different values according to the zones and regions of the prostate.

## Conclusion

In this feasibility study, we applied an in vivo electrical conductivity-based MR imaging method to image the differences in prostatic tissues. From the electrical conductivity images, we could quantify the tissue condition in terms of the conductivity distribution and anatomical significance. Conductivity images shown in this study indicated that the contrast between the prostatic tissues are distinguishable in a different way compared with conventional MR imaging techniques. Future studies will be focused on the verification and evaluation of conductivity differences between normal healthy men and patients with prostatic diseases.

## Data Availability

All authors had full access to all the data in the study and take responsibility for the integrity of the data and the accuracy of the data analysis. The datasets generated and/or analyzed during the current study are available from the corresponding author on reasonable request.

## Abbreviations

ECI: Electrical conductivity imaging
MR: Magnetic resonance
MREIT: Magnetic resonance electrical impedance tomography
ROI: Region of Interest
